# Supplementary material for: Kekveus brevisulcatus sp. nov., a new featherwing beetle from mid-Cretaceous amber of northern Myanmar (Coleoptera: Ptiliidae)
Source: PeerJ. 2023 May 11;11:e15306. doi: 10.7717/peerj.15306 (PMC10183168; doi:10.7717/peerj.15306)
Supplement: Supplemental Information 1 [file peerj-11-15306-s001.zip › Supporting Information.pdf]

Supplementary Information for

Kekveus brevisulcatus **sp. nov., a new featherwing beetle from mid-Cretaceous amber of northern Myanmar (Coleoptera: Ptiliidae)**

Yan-Da Li, Shûhei Yamamoto, Alfred F. Newton, Chen-Yang Cai

List of all Supplementary Information:

Figure S1. Placement of *Kekveus* within Ptiliidae, analyzed based on the matrix by Polilov et al. (2019a). Tree resulting from the constrained parsimony analysis under implied weights.

Figure S2. Relationship between *Kekveus brevisulcatus* and other Discheramocephalini, analyzed based on the matrix by Grebennikov (2009). Tree resulting from the constrained Bayesian analysis.

Data S1. List of characters used in the phylogenetic analyses based on Polilov et al. (2019a).

Data S2. List of characters used in the phylogenetic analyses based on Grebennikov (2009).

Data S3. Morphological datasets used for the analyses.

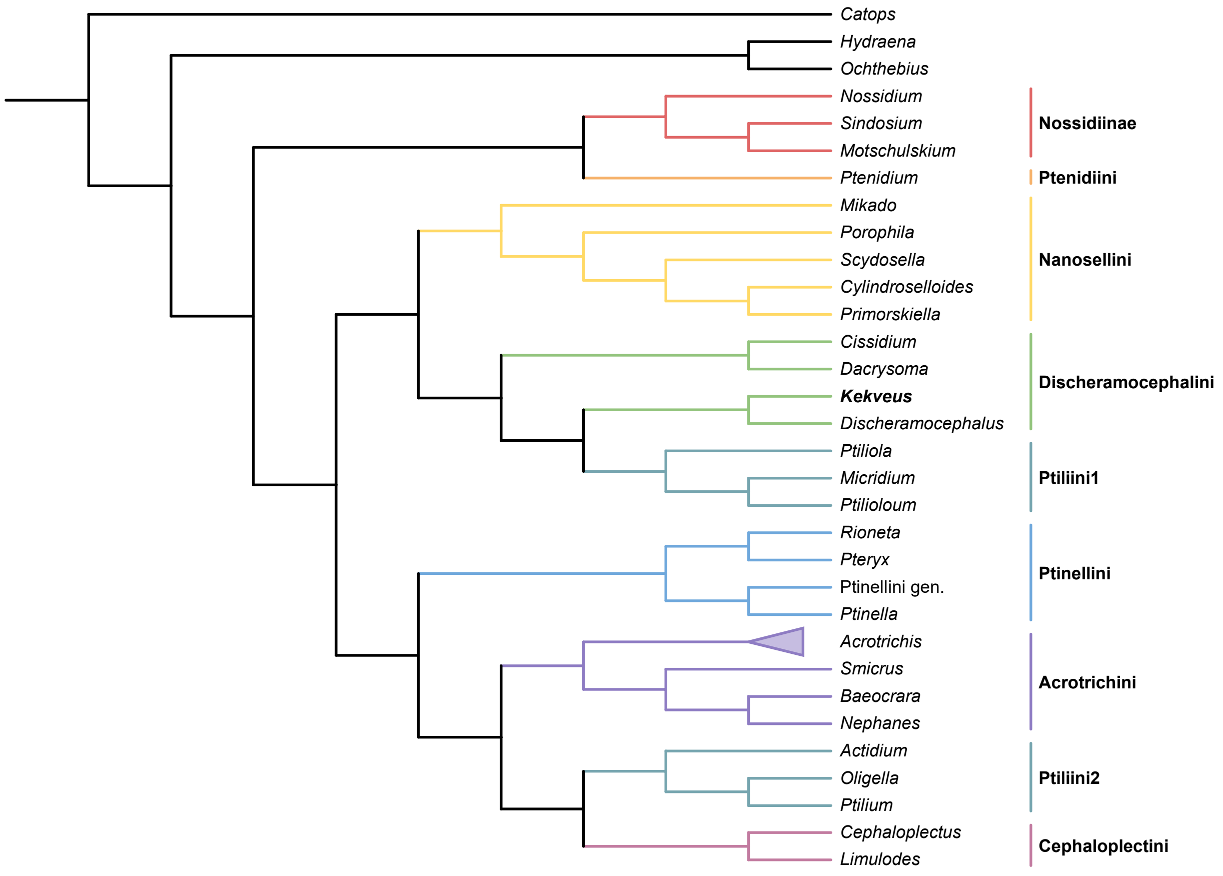

Figure S1. Placement of *Kekveus* within Ptiliidae, analyzed based on the matrix by Polilov et al. (2019a). Tree resulting from the constrained parsimony analysis under implied weights.

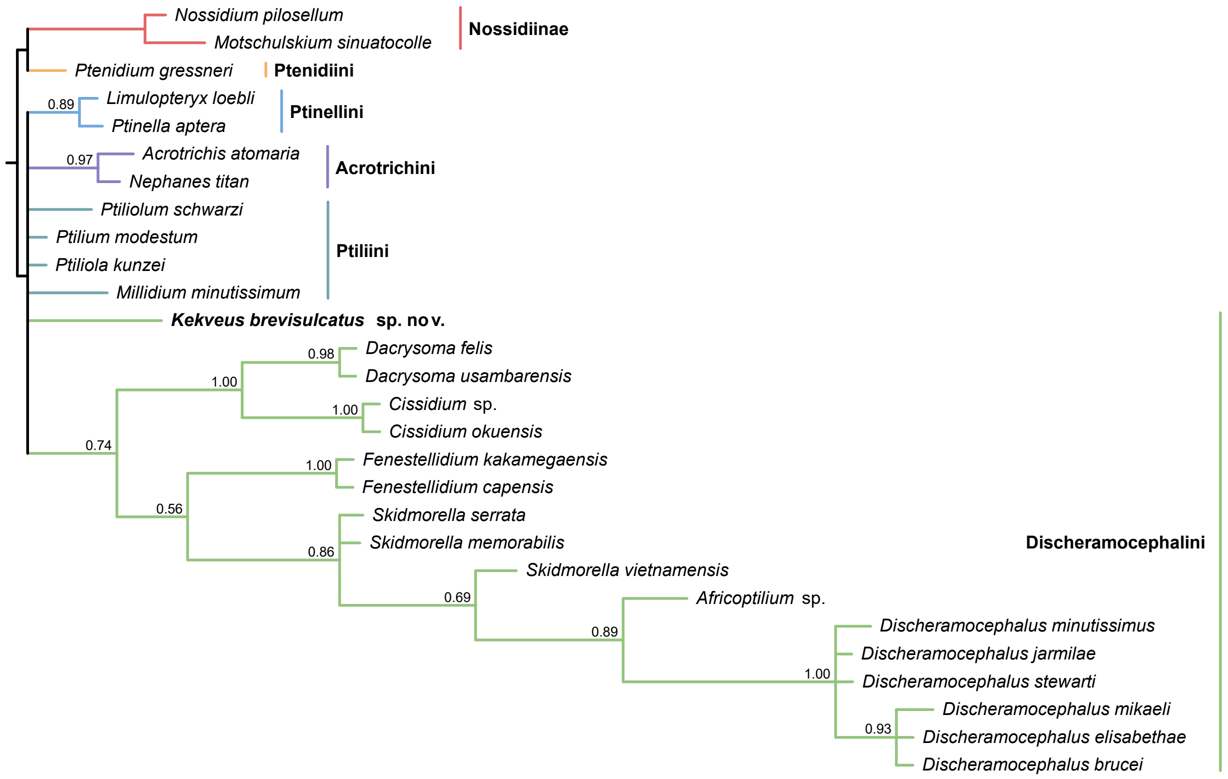

Figure S2. Relationship between *Kekveus brevisulcatus* and other Discheramocephalini, analyzed based on the matrix by Grebennikov (2009). Tree resulting from the constrained Bayesian analysis.

Data S1. List of characters used in the phylogenetic analyses based on Polilov et al. (2019a).

1 Orientation of head: (0) not distinctly deflexed; (1) strongly deflexed, frons and postclypeus in ventral position.

2 Epistomal ridge: (0) present; (1) absent.

3 Gular sutures: (0) present; (1) absent.

4 Transverse ventral genal bulge: (0) absent; (1) present.

5 Compound eyes: (0) present in all specimens; (1) dimorphic; (2) absent in all specimens.

6 Antennal grooves: (0) absent; (1) present, shallow; (2) present, distinct.

7 Number of antennomeres: (0) 11; (1) 10; (2) 9; (3) 8.

8 Insertion of flagellomere 1: (0) flagellomere 1 not retracted into pedicel; (1) flagellomere 1 distinctly retracted into pedicel and proximal flagellum distinctly narrower than the pedicel; (2) flagellomere 1 distinctly retracted into pedicel, flagellomere 1 and pedicel forming a continuous cylindrical structure.

9 Antennal club: (0) absent, antennae filiform or gradually widening distally; (1) 5-segmented; (2) 3-segmented (3) entire flagellum club-shaped, with short cylindrical flagellomeres.

10 Labral-mandibular locking mechanism: (0) absent; (1) present.

11 Shape of mandible: (0) apical part of mandibles distinct and prominent; (1) distal part shortened, not prominent; (2) distal part reduced.

12 Number of segments of galea: (1) two-segmented, with distinct suture; (2) one-segmented.

13 Shape of apical maxillary palpomere: (0) not aciculate; (1) aciculate.

14 Base of mentum: (0) separated from submentum by suture; (1) fused with submentum.

15 Shape of mentum: (0) wider than long, greatest width at base; (1) approximately as long as wide, or slightly longer than wide, with sides subparallel; (2) approximately as long as wide, dilated medially; (3) longer than wide, dilated in middle region.

16 Lateral premental lobes: (0) distinctly developed; (1) vestigial.

17 Palpiger: (0) separated from prementum; (1) fused.

18 Number of labial palpomeres: (0) 3; (1) 2; (2) 1.

19 Laminatentorium: (0) present; (1) absent.

20 Dorsal arms of tentorium: (0) present; (1) absent.

21 Cervical sclerites: (0) present; (1) absent.

22 Location of greatest width of pronotum: (0) in posterior half; (1) in middle region; (2) in anterior half.

23 Posterior pronotal angles: (0) not or only moderately produced posteriorly, not closely clinging to elytral humeri; (1) strongly prolonged and closely clinging to elytral humeri.

24 Microsculpture of dorsal surface of pronotum and elytra: (0) smooth or punctate; (1) distinctly reticulate or scaly.

25 Microsculpture of ventral surface of thoracic segments: (0) smooth or punctate; (1) distinctly reticulate or scaly; (2) dense vestiture of short hairs forming plastron.

26 Sculpture of pronotum: (0) smooth or finely sculptured; (1) with large rounded impressions; (2) with deep longitudinal impressions; (3) with large and deep impressions of irregular shape.

27 Pubescence of pronotum and elytra: (0) homogeneous; (1) with two types of hairs.

28 Hypomeral antennal pocket: (0) absent; (1) present.

29 Prothoracic ectodermal glands along lateral margins of prothorax: (0) absent; (1) present.

30 Prosternal process between procoxae: (0) present, moderately wide, at least partly separating procoxae; (1) present but narrow; (2) extremely short or absent, procoxae contiguous; (3) elongate and broad, extending beyond mesoventrite.

31 Extension of elytra: (0) covering entire dorsum of abdomen; (1) apical 2–3 abdominal tergites remaining uncovered.

32 Cuticular folds on inner elytral surface: (0) absent; (1) present.

33 Horizontally oriented deep fossa on each side of mesoventral keel: (0) absent; (1) present.

34 Mesopleuron: (0) separated from ventrite; (1) partly fused with ventrite; (2) completely fused.

35 Shape of meso- and metaventral processes between metacoxae: (0) mesoventral process ending near middle region of mesocoxae with distinct suture; (1) mesoventral process ending at posterior margin of mesocoxae with distinct suture; (2) mesoventral process projecting beyond posterior mesocoxal margin; (3) boundary between mesoventral process and metaventrite indiscernible, mesocoxae separated by narrow process; (4) boundary between mesoventral process and metaventrite discernible, with mesocoxae separated by wide process; (5) metaventral process wider than metacoxae, anteriorly directed, fused with mesoventral process.

36 Length of metaventrite: (0) longer than diameter of mesocoxae; (1) similar to diameter of mesocoxae.

37 Longitudinal impression along anterior margin of metaventrite: (0) absent; (1) present.

38 Metaventral lines: (0) absent; (1) present, reaching anterolateral angle of metaventrite; (2) present, reaching lateral margins of mesocoxae anteriorly.

39 Shape of metendosternite: (0) common stem short, arms long; (1) common stem widened and flattened, arms long; (2) common stem represented by thin wide transverse element, arms widely separated, long; (3) common stem short, arms long but thin and weakly sclerotized; (4) metendosternite compact, anterior arms short.

40 Apical muscular disc of arm of metendosternite: (0) present; (1) absent.

41 Length of alacrista: (0) not reaching beyond hind margin of metapostnotum; (1) distinctly reaching beyond hind margin of metapostnotum; (2) reaching beyond abdominal tergite III.

42 Single spur on each side of anterior region of metascutellum: (0) absent; (1) present.

43 Shape of metacoxae: (0) wider than long, contiguous or very narrowly separated; (1) small, distinctly separated; (2) enlarged, widening towards medial margin, contiguous, with wide metacoxal plates covering metafemora; (3) flattened, extending towards lateral margin, with cavity for retracted legs below them.

44 Shape of mesotrochanter: (0) unmodified; (1) narrowed, elongated.

45 Shape of femora: (0) not broadened and flattened; (1) distinctly broadened and flattened.

46 Number of tarsomeres: (0) five; (1) three.

47 Insertion of basal tarsomere: (0) not retracted into tibial apex; (1) retracted into tibial apex.

48 Shape of apical tarsomeres: (0) cylindrical; (1) dilated and flattened.

49 Size of pretarsal claws: (0) equal in size; (1) subequal.

50 Wings: (0) present; (1) dimorphic, present in some individual and absent in others; (2) absent.

51 Wing base: (0) membranous wing blade with veins; (1) petiole without membranous wing blade (Fig. 4).

52 Arrangement of folded wings below elytra: (0) overlapping; (1) parallel arrangement without overlap below elytra.

53 Ratio of width of wing blade and length of setae along its margin: (0) wing blade much wider than length of setae; (1) wing blade width similar to length of setae; (2) wing blade less than half as wide as length of setae.

54 Vein cubitus anterior (CuA): (0) present; (1) absent. According to Polilov et al. (2019a), here the nossidiine genera are coded as CuA present.

55 Number of veins in petiole or wing base: (0) three or more; (1) two; (2) one.

56 Number of veins in wing blade: (0) four or more; (1) three; (2) two.

57 Number of setae along margin of wing: (0) > 200; (1) 60–200; (1) < 60.

58 Wing folding patches (WFPs) on abdominal tergites: (0) absent; (1) present on tergites II–VII; (2) present on tergites II–VI; (3) present on tergites II–V.

59 WFPs: (0) absent; (1) represented by denticles; (2) represented by cuticular folds.

60 Shape of pterothorax and abdomen: (0) subparallel and posteriorly rounded; (1) distinctly triangular, tapering towards abdominal apex.

61 Position of abdominal segments VIII and IX: (0) retracted; (1) everted.

62 Shape of seventh visible sternite: (0) undivided; (1) divided into two lobes.

63 Hind margin of tergite X (pygidium): (0) without teeth or with few small teeth; (1) with 1–3 distinct apical teeth; (2) with specific flattened central tooth (sometimes bifurcated); (3) with two widely separated teeth and third tooth between them apically (the latter can be absent).

64 Spermathecal sperm pump: (0) absent; (1) present.

65 Shape of spermatheca: (0) strongly curved; (1) spherical; (2) funnel-shaped; (3) horseshoe-shaped, often asymmetrical; (4) ring-shaped; (5) poorly sclerotized, irregularly shaped; (6) simple helical; (7) complex helical.

66 Position of aedeagus: (0) shifted towards lateral body region; (1) placed along midline.

67 Parameres of aedeagus: (0) present; (1) strongly reduced; (2) absent.

68 Shape of aedeagus: (0) elongated and round in cross-section; (1) shortened and oval in cross-section; (2) shortened and flattened.

Data S2. List of characters used in the phylogenetic analyses based on Grebennikov (2009).

1 Body in cross-section, height/width ratio: (0) >0.85; (1) 0.65–0.85; (2) <0.55.

2 Body between pronotum and elytra in dorsal view: (0) not or only slightly constricted; (1) constricted.

3 Body behind pronotum: (0) not swollen laterally and vertically; (1) markedly swollen laterally and vertically.

4 Scale-like microsculpture on pronotum, elytra, and most of the body: (0) absent; (1) present.

5 Longitudinally oriented micro-ridges on ventral surface of prothorax: (0) absent; (1) present.

6 Elytral setae: (0) not forming clearly demarked longitudinal rows; (1) forming seven or eight longitudinal rows.

7 Transversely oriented deep depression behind eyes: (0) absent; (1) present, as two large fossae not connected by a groove; (2) present, as two large fossae connected by a groove; (3) present as deep groove extending laterad and ventrad.

8 Group of some 100–200 small punctures forming transverse band on head behind eyes: (0) absent; (1) present.

9 Apical antennomere: (0) not constricted at middle, not dumbbell-shaped; (1) constricted at middle, dumbbell-shaped.

10 Two longitudinal or anteriorly convergent grooves on mentum: (0) absent; (1) present.

11 Pronotosternal suture: (0) present; (1) absent.

12 Posterior edge of pronotum at middle: (0) straight or convex; (1) concave.

13 Depressions (not punctures bearing single setae) of pronotal disc: (0) absent; (1) rounded, present along posterior edge; (2) present as developed grooves of at least half pronotal length.

14 Longitudinal keel on scutellum: (0) absent; (1) present, either short (one-third of the length) or long and weak; (2) present, sharp, about as long as scutellum.

15 Transverse row of 12–13 round punctures on base of elytra and scutellum: (0) absent; (1) present.

16 Two deep exoskeletal fossae on scutellum: (0) absent; (1) present.

17 Meso-metaventral suture between mesocoxae: (0) clearly visible and forming a delimited border between sclerites; (1) obliterated, both sclerites fully amalgamated.

18 Meso-metaventral suture laterad of mesocoxae: (0) present and clearly visible externally as an externally projecting keel; (1) present only as internal thickening of cuticle, not visible externally.

19 Serration along meso-metaventral suture laterad of mesocoxae: (0) absent; (1) present.

20 Metaventral longitudinal lateral lines: (0) absent; (1) present.

21 Horizontally oriented perforation of mesoventral keel: (0) absent; (1) present.

22 Shape of horizontal perforation of mesoventral keel as visible in lateral view: (0) round; (1) vertically elongate, almost parallel-sided, about 2× longer than wide.

23 Grooves on mesoventrite originating from fossae of mesoventral keel and extending laterad: (0) absent; (1) present.

24 Fossae in anterior lateral corners of mesoventrite: (0) absent; (1) present.

25 Metacoxae: (0) separated by around one-fifteenth of metaventral width; (1) separated by around one-eighth of metaventral width; (2) separated by around one-sixth of metaventral width; (3) separated by around one-qurater of metaventral width.

26 Metacoxae: (0) not transverse, less than 2× as wide as long; (1) transverse, more than 2× as wide as long.

27 Posteriorly oriented projection of metaventral plate between metacoxae: (0) without two lateral teeth; (1) with two lateral teeth.

28 Alacrista of metathorax at middle: (0) without setae along margins; (1) with short setae along margins.

29 Metascutellar spur on alacrista: (0) absent; (1) present.

30 Hindwinge: (0) not or partly narrowed, individual seta of fringe not longer than width of wing blade; (1) narrowed, individual seta of fringe longer than width of wing blade.

31 Cavities on abdominal sternite VIII: (0) absent; (1) present.

32 Abdominal glands: (0) absent; (1) present.

33 Group of about 50–70 closely adjacent round micropores transversely oriented along posterior edge of tergite VIII: (0) absent; (1) present.

34 Transverse rows of teeth-like serration of abdominal sternites: (0) absent; (1) present.

35 Tergites XI and X: (0) free, separated from each other; (1) merged together into a single plate.

36 Single elongate internal sclerite parallel to aedeagus: (0) absent; (1) present.

37 Parameres: (0) present; (1) absent.
